# Real-World Tumor-Infiltrating Lymphocyte Therapy for Metastatic Melanoma: Treatment Delivery, Immune Reconstitution, and Cardiac Monitoring During High-Dose IL-2

**DOI:** 10.3390/curroncol33070379

**Published:** 2026-06-24

**Authors:** Mohamed A. Aboelatta, Jabra Zarka, Nika Tchatchua, Noureldin A. Aboelatta, Jeffrey E. Johnson, James W. Jakub, Justin Desroches, Justine Wilson-Miller, Anthony Tabiim, Deepti Behl, Heather N. Montane, Lisa A. Kottschade, Anastasios Dimou, Matthew S. Block, Elisabeth I. Heath, Bently Doonan, Mahesh Seetharam, Julian R. Molina, Jonathan E. Charnin, Paula Gill, Yi Lin, Binav Baral, Svetomir N. Markovic, Arkadiusz Z. Dudek

**Affiliations:** 1 Division of Medical Oncology, Mayo Clinic, Rochester, MN 55905, USA; 2 Division of Breast and Melanoma Surgical Oncology, Department of Surgery, Mayo Clinic, Rochester, MN 55905, USA; 3 Division of Surgical Oncology, Mayo Clinic, Jacksonville, FL 32224, USA; 4 Department of Pharmacy, Mayo Clinic, Rochester, MN 55905, USA; 5 Division of Hematology and Medical Oncology, Mayo Clinic, Jacksonville, FL 32224, USA; 6 Division of Medical Oncology, Mayo Clinic, Phoenix, AZ 85054, USA; 7 Department of Anesthesiology and Perioperative Medicine, Mayo Clinic, Rochester, MN 55905, USA; 8 Division of Hematology, Department of Medicine, Mayo Clinic, Rochester, MN 55905, USA

**Keywords:** tumor-infiltrating lymphocytes, adoptive cell therapy, metastatic melanoma, interleukin-2, immune reconstitution, cardiac toxicity, troponin, real-world outcomes

## Abstract

**Background/Objectives:** Tumor-infiltrating lymphocyte (TIL) therapy is an important option for patients with metastatic melanoma progressing after standard systemic therapy, but real-world data on treatment delivery, toxicity monitoring, and immune recovery remain limited. We evaluated clinical outcomes, treatment tolerance, immune reconstitution, and cardiac biomarker dynamics across three Mayo Clinic sites. **Methods:** We retrospectively analyzed adults with metastatic melanoma who received lymphodepleting chemotherapy followed by TIL infusion and high-dose interleukin-2 (IL-2) between April 2024 and December 2025. Clinical outcomes, treatment delivery, and adverse events were assessed. Longitudinal immune monitoring included CD4 and CD8 T-cell counts, CD4:CD8 ratio, and immunoglobulin G (IgG) at baseline and follow-up. In a prespecified cardiac sub-cohort, high-sensitivity troponin (hs-Tn) was measured during IL-2 administration to evaluate associations with cardiac events and IL-2 interruption. **Results:** Thirty-six patients underwent TIL infusion. The objective response rate was 50.0%, including complete responses in 13.9%, and the disease control rate was 72.2%. Median progression-free survival was 3.61 months, and median overall survival was 12.94 months. M1d disease was associated with inferior overall survival on univariable analysis (HR 6.55, 95% CI 2.03–21.17; *p* = 0.002), with attenuation after multivariable adjustment. Receipt of ≥3 IL-2 doses was associated with longer overall survival on univariable analysis (HR 0.20, 95% CI 0.06–0.64; *p* = 0.007), but this association also attenuated after adjustment. Longitudinal immune monitoring demonstrated persistent CD4 lymphopenia through 6 months, sustained inversion of the CD4:CD8 ratio, and declining IgG at months 3 and 6. In the cardiac sub-cohort (24 patients; 87 IL-2 doses), post-dose hs-Tn ≥15 ng/L was associated with clinically significant cardiac events (OR 9.6, 95% CI 1.5–60.6; *p* = 0.016) and IL-2 interruption (OR 3.4, 95% CI 1.1–10.7; *p* = 0.036). For cardiac events, hs-Tn ≥15 ng/L had 100% sensitivity and 100% negative predictive value. **Conclusions:** In routine practice, TIL therapy was feasible and active in metastatic melanoma. M1d disease identified a subgroup with poor survival, peri-dose hs-Tn showed promise as a tool to support safer IL-2 delivery, and prolonged CD4 suppression with IgG decline suggests that recovery after TIL therapy extends beyond initial hematologic reconstitution. These findings support prospective validation of biomarker-guided IL-2 monitoring and extended post-treatment immune surveillance.

## Simple Summary

Tumor-infiltrating lymphocyte therapy is a personalized immune cell treatment for people with advanced melanoma whose cancer has progressed after standard treatments. Although this therapy is now being used more often outside clinical trials, less is known about how it is delivered and monitored in routine practice. In this multisite Mayo Clinic study, we reviewed patients with metastatic melanoma who received this treatment and evaluated response, survival, immune recovery, interleukin-2 delivery, and heart monitoring with high-sensitivity troponin. The treatment produced meaningful responses, including complete responses in some patients. Patients with central nervous system metastatic disease had poorer survival. Immune recovery, especially CD4 T-cell recovery, remained delayed for several months. Troponin testing during interleukin-2 administration may help identify patients at low risk for clinically significant cardiac events.

## 1. Introduction

Advanced melanoma remains a major therapeutic challenge despite substantial progress with immune checkpoint blockade and BRAF/MEK-targeted therapy [1,2]. Even in the modern treatment era, a large proportion of patients with advanced melanoma do not experience durable benefit from available systemic therapies. Adoptive cell therapy with tumor-infiltrating lymphocytes (TIL) has emerged as an important strategy for this population. Early work from the Surgery Branch of the National Cancer Institute established the feasibility of TIL therapy administered with interleukin-2 (IL-2), and subsequent refinements incorporating nonmyeloablative lymphodepletion demonstrated that this approach could induce durable complete responses in heavily pretreated patients with metastatic melanoma [3,4,5]. These initial studies at NCI, together with additional institutional experiences using comparable lymphodepletion, TIL expansion, and IL-2-supported adoptive transfer procedures, established TIL therapy as one of the few cellular immunotherapy approaches capable of producing durable complete remissions in metastatic melanoma [6,7,8,9,10].

More recent studies have defined the contemporary role of TIL therapy in advanced melanoma. In the first randomized phase III trial, TIL therapy significantly improved progression-free survival compared with ipilimumab and produced higher objective response rates [11]. In parallel, lifileucel (Amtagvi), the first commercial TIL product, demonstrated clinical activity using a comparable treatment platform [12]. Long-term follow-up of the registrational C-144-01 study showed that lifileucel produced an objective response rate of 31.4%, a median duration of response of 36.5 months, and a 5-year overall survival rate of 19.7%, with responders tending to have lower tumor burden and fewer liver or brain metastases [13], leading to FDA approval of lifileucel [14]. However, TIL therapy remains clinically and operationally complicated [15]. The treatment requires patient evaluation and selection, tumor tissue procurement, ex vivo cell manufacturing, lymphodepleting chemotherapy, TIL infusion, and supportive high-dose IL-2. Expert consensus now emphasizes careful patient selection, use of bridging therapy when needed, and structured toxicity management during IL-2 administration [16]. At the same time, prior analyses have not demonstrated a clear association between the total number of IL-2 doses administered and clinical efficacy when IL-2 is abbreviated because of toxicity, underscoring that treatment tolerance may be as important as treatment intensity in real-world practice [17,18].

These issues are increasingly important as TIL therapy moves into broader clinical use, because successful implementation depends not only on antitumor activity but also on identifying patients at higher risk for poor outcomes, understanding the consequences of lymphodepleting therapy, and improving the safety of post-infusion IL-2 administration [16,18]. Real-world data addressing these questions remain limited, particularly with respect to treatment delivery, baseline metastatic disease characteristics, longitudinal immune reconstitution, and biomarker-guided assessment of cardiac toxicity. To address this problem, we performed a retrospective study across Mayo Clinic sites of patients with metastatic melanoma treated with TIL therapy to characterize treatment delivery and clinical outcomes, evaluate associations of baseline metastatic disease characteristics and IL-2 exposure with survival, define immune reconstitution after lymphodepleting chemotherapy, and assess whether peri-dose high sensitivity troponin (hs-Tn) identifies clinically significant cardiac events and interruption of IL-2 therapy.

## 2. Materials and Methods

### Study Design and Patients

We conducted a retrospective multisite cohort study of adult patients with metastatic melanoma who underwent TIL infusion at Mayo Clinic sites between April 2024 and December 2025. The primary analytic cohort included all infused patients, including those who received out-of-specification (OOS) TIL product under single-patient Investigational New Drug (IND) authorization. Separate immune reconstitution and cardiac biomarker sub-cohorts were defined according to availability of longitudinal immune monitoring data and paired peri-dose hs-Tn measurements, respectively. The study was approved by the Mayo Clinic Institutional Review Board [IRB 25-004730], with a waiver of informed consent in accordance with institutional policy.

Clinical data were abstracted from the electronic medical record, including age, sex, Eastern Cooperative Oncology Group (ECOG) performance status, melanoma subtype, metastatic stage, sites of metastases, baseline laboratory values, baseline cardiac testing, prior systemic therapy lines, bridging therapy, treatment timelines, IL-2 delivery, radiographic response, phase-attributed treatment-emergent toxicities, and infections. Baseline metastatic stage and sites of disease involvement at the time of TIL infusion, including liver, bone and brain metastases, were recorded. Survival analyses evaluating baseline disease extent focused on M1d disease. Bridging therapy was defined as anticancer treatment administered after tumor procurement and before lymphodepleting chemotherapy to maintain disease control during product manufacturing. It was administered at the treating physician’s discretion and included radiotherapy and systemic therapies.

Tumor tissue was obtained by surgical resection or biopsy of a clinically appropriate lesion at the discretion of the treating surgical and medical oncology teams. Patients received nonmyeloablative lymphodepletion with cyclophosphamide 60 mg/kg IV daily with mesna for 2 days followed by fludarabine 25 mg/m^2^ IV daily for 5 days, then TIL infusion and inpatient high-dose (600,000 IU/kg) IL-2 with intensive care unit monitoring. IL-2 was held or discontinued at treating physician discretion for clinically significant hemodynamic instability, hypoxia, renal dysfunction, arrhythmia, electrocardiographic abnormality, cardiac biomarker concern, infection, or other organ toxicity. Out-of-specification product classification was assigned according to commercial manufacturing and release documentation. Available institutional manufacturing and regulatory records were reviewed to identify the documented reason for OOS classification and whether infusion occurred under single-patient IND authorization. Proprietary manufacturer-defined release thresholds, infused viable cell dose, and complete commercial release documentation were not uniformly available for independent adjudication.

Clinical outcomes included best overall response (BOR), objective response rate (ORR), disease control rate (DCR), OS, progression-free survival (PFS), and treatment-related toxicities. Best overall response (BOR) was categorized as complete response, partial response, stable disease, or progressive disease based on RECIST v1.1 [19] in routine clinical practice. Objective response rate (ORR) was defined as the proportion of patients achieving complete or partial response, and disease control rate (DCR) as the proportion achieving complete response, partial response, or stable disease. OS and PFS were measured from the date of TIL infusion. Treatment-emergent adverse events were abstracted from the electronic medical record and graded using CTCAE v5.0. Events were categorized according to the dominant treatment phase during which they occurred: tumor harvest, lymphodepleting chemotherapy, the interval after TIL infusion and before the first IL-2 dose, and IL-2 administration. Because attribution in a multicomponent cellular therapy regimen is challenging retrospectively, events were summarized as phase-attributed treatment-emergent toxicities. Treatment-related mortality was assessed separately.

Immune reconstitution outcomes included absolute CD4 and CD8 T-cell counts, CD4:CD8 ratio, and immunoglobulin G (IgG) measured at baseline and approximately 1, 3, and 6 months after TIL infusion. Clinically significant CD4 lymphopenia was defined as CD4 <200 cells/µL and summarized descriptively at each time point.

Cardiac outcomes were evaluated in a prespecified sub-cohort of patients treated at Mayo Clinic Rochester, where paired pre-dose and post-dose hs-Tn measurements were obtained routinely during IL-2 administration as part of institutional monitoring practice, including in asymptomatic patients. This monitoring approach was not routinely used at the Scottsdale or Jacksonville sites. Clinically significant cardiac events were defined as any of the following occurring after an IL-2 dose: QTc prolongation >480 ms, clinically significant arrhythmia excluding isolated sinus tachycardia, new electrocardiographic abnormality, vasopressor requirement, or cardiology consultation. Subsequent IL-2 dose interruption was defined as holding the next planned IL-2 dose.

Continuous variables were summarized as median with interquartile range (IQR), and categorical variables as number with percentage. Kaplan–Meier methods were used to estimate OS and PFS, with group comparisons by log-rank testing and hazard ratios (HRs) estimated using Cox proportional hazards models. Given the modest sample size, multivariable survival models were intentionally parsimonious and limited to clinically selected covariates. Potential sources of bias were addressed by including all consecutive infused patients during the study period, defining analytic sub-cohorts a priori according to data availability, and limiting adjusted models to clinically selected variables to reduce overfitting. No imputation was performed; analyses were conducted using available data, and the number of observations contributing to immune and cardiac analyses was reported. Longitudinal immune reconstitution was analyzed using generalized estimating equations (GEE) with baseline as the reference time point. For cardiac analyses, paired pre-dose and post-dose hs-Tn values were compared using the Wilcoxon signed-rank test. Dose-level associations between post-dose hs-Tn and cardiac events or subsequent IL-2 interruption were evaluated using nonparametric testing and clustered logistic regression to account for repeated IL-2 doses within patients. Diagnostic performance characteristics for post-dose hs-Tn ≥15 ng/L were calculated for clinically significant cardiac events and subsequent IL-2 dose interruption. All tests were two-sided, and *p* < 0.05 was considered statistically significant. Analyses were performed using R version 4.5.1 (R Foundation for Statistical Computing, Vienna, Austria).

## 3. Results

### 3.1. Patient Characteristics and Treatment Delivery

Between April 2024 and December 2025, 45 patients underwent tumor procurement across Mayo Clinic sites. Thirty-six (80%) ultimately received TIL infusion and comprised the primary analytic cohort. Ten of forty-five (22.2%) manufactured products were classified as out-of-specification, seven (70.0%) of which were infused under single-patient IND authorization, representing 19.4% of the 36 patient infused cohort. Among all OOS products, the documented reasons in available institutional records were low cell count in nine (90.0%) patients and low CD8/CD4 ratio in one (10.0%) patient. Among infused OOS products, the documented reasons were low cell count in 6 (85.7%) patients, and low CD8/CD4 ratio in one (14.3%) patient. Median age at infusion was 60 years (Interquartile Range [IQR] 50–70), 21 patients (58.3%) were male, and most had ECOG performance status 0 (31/36, 86.1%). Melanoma subtype was cutaneous in 27 patients (75.0%), mucosal in 8 (22.2%) and acral in 1 (2.8%). At infusion, 23 patients (63.9%) had M1c disease and 8 (22.2%) had M1d disease. Liver metastases were present in 11 (30.6%), brain metastases in 5 (13.9%), and bone metastases in 13 (36.1%). Baseline LDH exceeded the upper limit of normal in 14 patients (38.9%). Patients had received a median of 3 (IQR 2–4) lines of therapy prior to TIL treatment. Bridging therapy was administered in 20 patients (55.6%), most commonly radiotherapy (10/36, 27.8%). Bridging categories were non-mutually exclusive because one patient received more than one bridging modality. Among patients receiving bridging therapy, the median interval from bridging therapy to hospitalization was 24 days (range: 6–36), and the median interval from bridging therapy to TIL infusion was 32 days (range: 13–51). Median tissue procurement-to-infusion interval was 50 days (IQR 49–55), and median infusion-to-first IL-2 interval was 7.8 h (IQR 6.2–20.3). Patients received a median of 4 IL-2 doses (IQR 3–5), and 29 of 36 (80.6%) received ≥3 doses (Table 1).

### 3.2. Clinical Outcomes

**Figure 1:**
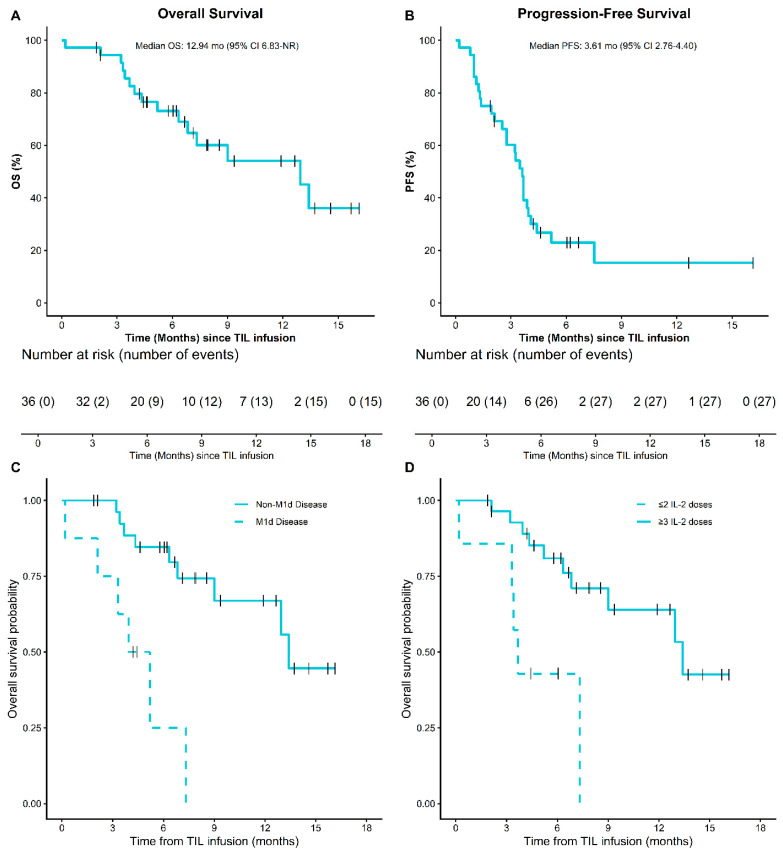
Kaplan–Meier survival curves from TIL infusion. (**A**) Overall survival; median OS 12.94 months (95% CI 6.83–NR). (**B**) Progression-free survival; median PFS 3.61 months (95% CI 2.76–4.40). (**C**) Overall survival according to presence of M1d disease at infusion (Yes vs. No); median OS 4.57 months (95% CI 3.32–NR) versus 13.40 (95% CI 9.00–NR), log-rank *p* < 0.001. (**D**) Overall survival according to IL-2 dose exposure (≤2 vs. ≥3 doses); median OS 3.65 months (95% CI 3.32–NR) versus 13.40 months (95% CI 9.00–NR), log-rank *p* = 0.003.

BOR included complete response in 5 patients (13.9%), partial response in 13 (36.1%), stable disease in 8 (22.2%), and progressive disease in 10 (27.8%), yielding an ORR of 50.0% and a DCR of 72.2% (Table 2). Median OS was 12.94 months (95% Confidence Interval [CI] 6.83–NR), and median PFS was 3.61 months (95% CI 2.76–4.40) (Table 2; Figure 1A,B). Median follow-up was 8.54 months (95% CI 6.67–14.59). Patients receiving ≥3 IL-2 doses had a numerically higher ORR than those receiving ≤2 doses (55.2% vs. 28.6%), although this difference was not statistically significant (odds ratio [OR] 3.08, 95% CI 0.51–18.54; *p* = 0.402) (Supplemental Table S1).

### 3.3. Factors Associated with Survival

M1d disease was associated with inferior OS (median: 4.57 months vs. 13.40, *p* <0.001) (Figure 1C). On univariable Cox analysis, M1d disease showed a directionally adverse association with OS (Hazard Ratio [HR] 6.55, 95% CI 2.03–21.17; *p* = 0.002), which attenuated in a parsimonious multivariable model (HR 4.23, 95% CI 0.98–18.20; *p* = 0.053). By contrast, receipt of ≥3 IL-2 doses was associated with improved OS (median: 13.40 vs. 3.65 months; *p* = 0.003) (Figure 1D). This association was significant on univariable analysis (HR 0.20, 95% CI 0.06–0.64; *p* = 0.007) but was attenuated after multivariable adjustment (HR 0.47, 95% CI 0.11–2.02; *p* = 0.309). Product out-of-specification status was not significantly associated with OS (HR 2.03, 95% CI 0.63–6.57; *p* = 0.238), nor was bridging therapy. Higher continuous CRP was associated with worse OS on univariable analysis (Table 3). Given the small number of infused OOS products and limited granularity regarding manufacturer-defined OOS criteria, outcomes according to OOS status were summarized descriptively and were not used for inferential survival modeling. For PFS, no statistically significant associations were observed for M1d disease, bridging therapy, or IL-2 dose category (Supplemental Table S2).

### 3.4. Immune Reconstitution After TIL Therapy

Longitudinal CD4, CD8, and CD4:CD8 ratio data were available for 24 patients (66 total observations), and IgG data were available for 17 patients (37 observations). Relative to baseline, CD4 counts remained significantly suppressed at month 1 (regression coefficient (B) −340.4, 95% CI −523.6 to −157.2; *p* < 0.001), month 3 (B −396.4, 95% CI −560.5 to −232.2; *p* < 0.001), and month 6 (B −432.2, 95% CI −611.0 to −253.4; *p* < 0.001). In contrast, CD8 counts increased at month 1 (B +503.2, 95% CI 52.6–953.9; *p* = 0.029) and month 3 (B +243.5, 95% CI 42.8–444.2; *p* = 0.017), but not at month 6 (*p* = 0.744). The CD4:CD8 ratio remained significantly reduced at all follow-up time points, consistent with persistent inversion of the ratio. IgG levels were not significantly changed at month 1 but declined at month 3 (B −172.7, 95% CI −308.1 to −37.3; *p* = 0.012) and month 6 (B −237.8, 95% CI −395.9 to −79.8; *p* = 0.003) (Table 4; Supplemental Table S3). No patients received IVIG supplementation and there was no correlation between IgG nadir and infection events. Prophylactic antibiotics were provided to all patients and not based on IgG levels.

Clinically significant CD4 lymphopenia remained common throughout follow-up. CD4 <200 cells/µL was observed in 12 of 20 patients (60%) at month 1, 13 of 16 (81%) at month 3, and 9 of 10 (90%) at month 6. Although infections occurred during follow-up, comparisons of infection rates by CD4 category were descriptive and underpowered because of small numbers and substantial missingness at later time points.

### 3.5. Peri-Dose High-Sensitivity Troponin, Cardiac Events, and IL-2 Dose Interruption

A cardiac biomarker sub-cohort of 24 patients contributed paired pre-dose and post-dose hs-Tn measurements across 87 evaluable IL-2 doses. Sixteen patients (67%) had at least one post-dose hs-Tn value ≥15 ng/L, and 48 of 87 doses (52%) were followed by a post-dose hs-Tn ≥15 ng/L. Post-dose hs-Tn values were significantly higher than paired pre-dose values overall (*p* = 0.023). Eight of 24 patients (33%) experienced at least one clinically significant cardiac event (defined as QTc prolongation, clinically significant arrhythmia, new electrocardiographic abnormality, vasopressor requirement, or cardiology consultation); no post-IL-2 reduction in left ventricular ejection fraction was observed (Supplemental Table S4).

In dose-level analyses, clinically significant cardiac events occurred only after doses with post-dose hs-Tn ≥15 ng/L (15/48 doses, 31%) and were not observed after doses with post-dose hs-Tn ≤14 ng/L (0/39 doses; *p* < 0.001). Higher post-dose hs-Tn was also associated with holding the next planned IL-2 dose (*p* < 0.001). In clustered logistic regression accounting for repeated doses within patients, post-dose hs-Tn ≥15 ng/L was associated with cardiac events (OR 9.6, 95% CI 1.5–60.6; *p* = 0.016) and subsequent IL-2 dose interruption (OR 3.4, 95% CI 1.1–10.7; *p* = 0.036). When modeled continuously, each 1-unit increase in log-transformed post-dose hs-Tn was associated with higher odds of cardiac events (OR 3.25, 95% CI 1.59–6.65; *p* = 0.001) and IL-2 dose interruption (OR 2.82, 95% CI 1.63–4.88; *p* < 0.001) (Table 5). Using a post-dose hs-Tn threshold of ≥15 ng/L, sensitivity, specificity, positive predictive value, and negative predictive value for clinically significant cardiac events were 100.0%, 54.2%, 31.3%, and 100.0%, respectively. For subsequent IL-2 dose interruption, corresponding values were 77.3%, 54.7%, 37.0%, and 87.5%, respectively (Supplemental Table S5).

### 3.6. Treatment-Emergent Toxicities Across the TIL Treatment Course

Tumor harvest was associated with one allergic reaction (2.8%) and no grade ≥3 harvest complications. During lymphodepleting chemotherapy, common events included nausea (52.8%), diarrhea (38.9%), fluid overload or edema (22.2%), fatigue or malaise (22.2%), and vomiting (16.7%); grade ≥3 events during lymphodepletion were infrequent. After TIL infusion and before the first IL-2 dose, chills or rigors and tachycardia each occurred in 4 patients (11.1%), and neutropenic fever or bacteremia occurred in 3 patients (8.3%), including two cases with culture-positive bacteremia. During IL-2 administration, grade ≥3 toxicities were most frequently thrombocytopenia (77.8%), neutropenic fever (58.3%), hypoxia (22.2%), hypotension (11.1%), transaminitis (11.1%), hypophosphatemia (11.1%), and acute kidney injury (8.3%); platelet transfusions were required in 22 patients (61.1%). No treatment-related deaths occurred (Table 6).

## 4. Discussion

In this real-world Mayo Clinic cohort, TIL therapy demonstrated clinically meaningful activity in patients with metastatic melanoma while also providing insight into several practical issues that are increasingly relevant as this treatment moves into broader use. Notably, the cohort included the first systematic reporting of patients treated with out-of-specification product under single-patient IND authorization, reflecting real-world delivery of TIL therapy beyond idealized trial conditions. We observed an objective response rate of 50.0%, a complete response rate of 13.9%, and median overall survival of 12.9 months. Beyond efficacy, our data highlight four clinically relevant observations: baseline M1d disease was associated with inferior overall survival, receipt of ≥3 IL-2 doses was associated with improved overall survival in univariable analysis but attenuated after multivariable adjustment, serial peri-dose hs-Tn identified patients at risk for cardiac events and interruption of IL-2, and prolonged CD4 lymphopenia with sustained inversion of the CD4:CD8 ratio persisted through six months after treatment. Together, these findings help define how TIL therapy is delivered, monitored, and followed in routine clinical practice.

Our efficacy results are broadly consistent with contemporary TIL studies. In the randomized phase III trial by Rohaan et al., TIL therapy achieved an objective response rate of 49% and significantly prolonged progression-free survival compared with ipilimumab (7.2 vs. 3.1 months) [11]. In the registrational lifileucel program, pooled analysis of C-144-01 demonstrated clinically meaningful activity in heavily pretreated patients, and 5-year follow-up showed an objective response rate of 31.4%, median duration of response of 36.5 months, and 5-year overall survival of 19.7% [13,20]. Meta-analyses have likewise confirmed durable clinical benefit across TIL studies in advanced cutaneous melanoma [17]. In the 2019 meta-analysis by Dafni et al., the pooled ORR in the high-dose IL-2 cohort was 43% (n = 332), whereas the updated 2024 meta-analysis by Martin-Lluesma et al. reported an ORR of 44% in the corresponding high-dose IL-2 cohort (n = 434) [17,21]. Against this backdrop, the response rate observed in our cohort is reassuring and supports the feasibility of TIL delivery in routine practice, even though survival outcomes remain constrained by the adverse disease biology typical of patients referred for cellular therapy. The adverse association we observed between M1d disease and overall survival is also directionally consistent with prior reports showing that responders to lifileucel tend to have lower tumor burden and fewer liver or brain metastases, and with broader evidence linking higher disease burden to worse outcomes after TIL therapy [13,21].

The contribution of post-infusion IL-2 remains difficult to disentangle from patient fitness and treatment tolerance. In our cohort, patients who received ≥3 IL-2 doses had a numerically higher objective response rate and significantly longer overall survival on univariable analysis, although this association attenuated after adjustment for baseline covariates. We interpret this cautiously. The ability to receive additional IL-2 doses likely reflects both adequate treatment delivery and greater physiologic reserve. At the same time, we are hesitant to conclude that very abbreviated IL-2 exposure is fully equivalent to receiving several doses. Contemporary studies have reported activity with attenuated IL-2 regimens [22,23,24], and expert guidance emphasizes prioritizing safety when IL-2 must be discontinued for toxicity [16]. Taken together, these findings suggest that the ability to receive at least several IL-2 doses may serve as a descriptive benchmark of treatment tolerance in routine practice rather than a prescriptive dosing target. Real-time clinical tolerance should remain the primary determinant of dosing [25]. Prospective studies will be needed to determine whether biomarker-guided or toxicity-adapted IL-2 de-escalation can preserve efficacy while reducing morbidity.

A particularly actionable observation in this study was the relationship between peri-dose hs-Tn elevation and clinically significant cardiac events during IL-2 administration. Cardiovascular toxicity during TIL therapy has been described previously, including clinically important arrhythmias, myocarditis-like presentations, and treatment-related mortality, but objective tools to guide risk stratification during IL-2 remain incompletely defined [26,27]. In our cohort, cardiac events occurred exclusively following IL-2 doses associated with elevated post-dose hs-Tn, and a threshold ≥15 ng/L demonstrated high sensitivity and negative predictive value for clinically significant cardiac events. Although troponin elevation in this setting is likely multifactorial and the positive predictive value was modest, these findings suggest that serial hs-Tn monitoring may serve as a useful adjunctive biomarker for risk stratification during IL-2 administration. In practice, elevated hs-Tn levels may help prompt closer telemetry review, electrocardiographic reassessment, or cardio-oncology consultation when decisions regarding additional IL-2 dosing are being considered.

A second major contribution of this study is the longitudinal characterization of immune recovery after TIL therapy. Prior TIL studies and consensus reports have understandably focused on acute toxicities and early recovery, and in the 5-year C-144-01 analysis most grade 3/4 cytopenias resolved to grade ≤2 by day 30 [13,28]. Our data show that later immune reconstitution follows a different trajectory. CD4 counts remained markedly suppressed through six months, the CD4:CD8 ratio remained inverted throughout follow-up, and IgG declined at later time points. These findings suggest that hematologic recovery does not necessarily reflect immune recovery. Fludarabine-based lymphodepletion has long been recognized to modulate endogenous immunity and facilitate persistence of adoptively transferred T cells [29,30,31,32], but the downstream consequence in routine TIL practice may be a prolonged helper T-cell deficit that is underappreciated when follow-up is limited to the immediate post-treatment period.

This study has several limitations. It was retrospective and relatively small, which limits statistical power and increases the risk of residual confounding. The cohort was assembled across Mayo Clinic sites within a single health system, which improves consistency of care pathways but may limit generalizability. Survival analyses were confined to infused patients and therefore do not address attrition during manufacturing or pre-infusion selection. Detailed dose-, cycle-, and response-level data for all prior systemic therapies were not uniformly available, particularly for therapies administered outside the Mayo Clinic system, limiting granular assessment of pre-TIL treatment history and washout intervals. Proprietary manufacturer-defined release thresholds, complete product-level OOS documentation, and infused viable TIL cell dose were not uniformly available for independent adjudication. As a result, OOS product characteristics were summarized descriptively rather than incorporated into inferential survival models. IL-2 dose delivery is intrinsically susceptible to confounding by treatment tolerance and survivorship bias. Longitudinal immune monitoring was incomplete at later time points, particularly for IgG, and the cardiac biomarker analysis was limited to the Rochester subgroup where routine peri-dose hs-Tn monitoring was performed. In addition, response assessment reflected routine clinical practice rather than central blinded review. For these reasons, our findings should be viewed as hypothesis-generating.

## 5. Conclusions

Despite these limitations, the study has immediate practical relevance. As commercial TIL therapy moves from specialized trial settings into broader routine care, clinicians need data not only on response but also on who does poorly, how much IL-2 patients actually receive, how to monitor for cardiac toxicity, and how long immune perturbations persist after treatment. Our results suggest that baseline metastatic pattern, particularly M1d disease, may inform counseling and referral timing; serial hs-Tn may help identify patients at low versus higher risk for clinically significant cardiac events during IL-2 administration; and prolonged CD4 suppression may warrant follow-up beyond hematologic recovery alone. These questions are best addressed in prospective multicenter cohorts, but they are already relevant to day-to-day TIL practice.

## Figures and Tables

**Table 1:** Baseline Characteristics.

Variable	Overall (N = 36)
**Demographics and baseline clinical status**	
Age at melanoma diagnosis, years	53 (IQR 46–63)
Age at TIL infusion, years	60 (IQR 50–70)
Age ≥65 at TIL infusion, n (%)	11 (30.6%)
Male sex, n (%)	21 (58.3%)
ECOG 0, n (%)	31 (86.1%)
ECOG 1, n (%)	5 (13.9%)
**Melanoma subtype and disease extent at infusion**	
Melanoma subtype, n (%)	
Cutaneous	27 (75.0%)
Mucosal	8 (22.2%)
Acral	1 (2.8%)
AJCC metastatic stage at TIL infusion, n (%)	
M1a	2 (5.6%)
M1b	3 (8.3%)
M1c	23 (63.9%)
M1d	8 (22.2%)
Liver metastases at TIL infusion, n (%)	11 (30.6%)
Brain metastases at TIL infusion, n (%)	5 (13.9%)
Bone metastases at TIL infusion, n (%)	13 (36.1%)
**Baseline laboratory and cardiac variables**	
Baseline labs	
LDH, U/L	211 (IQR 178–283)
LDH > ULN, n (%)	14 (38.9%)
CRP, mg/L	16 (IQR 5–44)
Ferritin, ng/mL	426 (IQR 235–647)
Baseline hs-Tn, ng/L	10 (IQR 7–18)
hs-Tn ≥15 ng/L, n (%)	6 (16.7%)
Baseline LVEF, %	63 (IQR 58–64)
**Prior therapy and bridging therapy**	
Prior systemic therapy lines, median (IQR)	3 (IQR 2–4)
Bridging therapy, n (%)	20 (55.6%)
Radiotherapy	10 (27.8%)
PD-1/PD-L1 inhibitor	3 (8.3%)
PD-1 + CTLA-4 inhibitor	2 (5.6%)
Chemotherapy	2 (5.6%)
PD-1/PD-L1 inhibitor + Chemotherapy	1 (2.8%)
BRAFi/MEKi	1 (2.8%)
Other (nilotinib, imatinib)	2 (5.6%)
Bridging therapy to hospitalization, days, median (range)	24 (6–36)
Bridging therapy to TIL infusion, days, median (range)	32 (13–51)
**Manufacturing and treatment delivery**	
Patients undergoing tumor procurement, n	45
Patients receiving TIL infusion, n/N (%)	36/45 (80.0%)
OOS product among procured/manufactured patients, n/N (%)	10/45 (22.2%)
Infused OOS product, n/N (%)	7/36 (19.4%)
Infused OOS reason: low cell count	6/7 (85.7%)
Infused OOS reason: low CD8/CD4 ratio	1/7 (14.3%)
Harvest-to-infusion interval, days	50 (IQR 49–55)
Infusion to first IL-2 dose, hours	7.8 (IQR 6.2–20.3)
IL-2 doses received	4 (IQR 3–5)
IL-2 ≥3 doses, n (%)	29 (80.6%)

AJCC, American Joint Committee on Cancer; CTLA-4, cytotoxic T-lymphocyte-associated protein 4; ECOG, Eastern Cooperative Oncology Group; hs-Tn, high-sensitivity troponin; IQR, interquartile range; LDH, lactate dehydrogenase; ULN, upper limit of normal (222 U/L); PD-1, programmed cell death protein 1; PD-L1, programmed death-ligand 1; M1a, distant skin, subcutaneous, or nodal metastases; M1b, lung metastases; M1c, non-CNS visceral metastases; M1d, central nervous system metastases; BRAFi/MEKi, BRAF and MEK inhibitors; Bridging modality categories were not mutually exclusive, one patient received a combination of ICI and imatinib.

**Table 2:** Clinical Outcomes after TIL Therapy.

Response Category	N (%)	Median (Months) (95% CI)
Complete Response (CR)	5 (13.9%)	–
Partial Response (PR)	13 (36.1%)	–
Stable Disease (SD)	8 (22.2%)	–
Progressive Disease (PD)	10 (27.8%)	–
Objective Response Rate (CR + PR)	18 (50.0%)	–
Disease Control Rate (CR + PR + SD)	26 (72.2%)	–
Overall Survival (months)	–	12.94 (95% CI 6.83–NR)
Progression-Free Survival (months)	–	3.61 (95% CI 2.76–4.40)

**Table 3:** Univariate and Multivariable Cox Regression Analyses of Factors Associated with Overall Survival.

Variable (Comparison)	Univariate HR (95% CI)	*p*	Multivariate HR (95% CI)	*p*
**Demographics and clinical status**				
Sex (Female vs. Male)	1.19 (0.39–3.57)	0.762	–	-
Age at TIL (<65 vs. ≥65 years)	0.59 (0.17–2.12)	0.422	-	-
ECOG Performance Status (≥1 vs. 0)	1.58 (0.63–8.54)	0.203	-	-
Melanoma subtype (Cutaneous vs. Mucosal)	0.96 (0.26–3.49)	0.946	-	-
**Disease extent**				
M1d (Yes vs. No)	6.55 (2.03–21.17)	0.002	4.23 (0.98–18.20)	0.053
Liver mets (Yes vs. No)	1.69 (0.60–4.80)	0.321	-	-
Bone mets (Yes vs. No)	0.82 (0.26–2.59)	0.730	-	-
**Baseline laboratory variables**				
LDH > ULN (Yes vs. No)	1.37 (0.49–3.87)	0.550	-	-
LDH (per 100 U/L increase)	1.19 (0.97–1.47)	0.101	-	-
CRP > ULN (Yes vs. No)	3.57 (0.79–16.19)	0.100	-	-
CRP (per 10 mg/L increase)	1.20 (1.00–1.43)	0.046	-	-
Ferritin > ULN (Yes vs. No)	0.48 (0.15–1.52)	0.212	-	-
**Treatment delivery variables**				
TIL infusion to IL-2 first dose interval (≤8 h vs. >8 h)	1.68 (0.51–5.58)	0.395	-	-
Bridging therapy (Yes vs. No)	1.15 (0.41–3.25)	0.787	-	-
IL-2 doses (≥3 vs. ≤2)	0.20 (0.06–0.64)	0.007	0.47 (0.11–2.02)	0.309

CI, confidence interval; HR, hazard ratio; ECOG, Eastern Cooperative Oncology Group performance status; ULN, upper limit of normal; LDH, lactate dehydrogenase; CRP, C-reactive protein; TIL, tumor-infiltrating lymphocyte; IL-2, interleukin-2; ULN for LDH = 222 U/L; ULN for CRP = 5 mg/L; ULN for ferritin = 175 ng/mL; OOS, out-of-specification product defined per industrial manufacturing criteria.

**Table 4:** Longitudinal Immune Reconstitution after TIL infusion.

Outcome	Month	B (vs. Baseline)	95% CI	*p*
**CD4**	M1	−340.395	−523.555 to −157.235	<0.001
	M3	−396.369	−560.505 to −232.232	<0.001
	M6	−432.223	−611.038 to −253.408	<0.001
**CD8**	M1	+503.235	52.584 to 953.885	0.029
	M3	+243.454	42.752 to 444.156	0.017
	M6	+27.836	−139.502 to 195.173	0.744
**CD4:CD8 Ratio**	M1	−1.943	−2.507 to −1.379	<0.001
	M3	−1.923	−2.468 to −1.379	<0.001
	M6	−1.864	−2.411 to −1.317	<0.001
**IgG**	M1	−57.586	−219.577 to 104.405	0.486
	M3	−172.680	−308.108 to −37.251	0.012
	M6	−237.848	−395.868 to −79.828	0.003

B, beta coefficient (regression coefficient); M, month; CI, confidence interval; CD4, absolute CD4^+^ T-cell count (cells/µL); CD8, absolute CD8^+^ T-cell count (cells/µL); IgG, immunoglobulin G (mg/dL).

**Table 5:** Association of Post-Dose hs-Tn with Cardiac Events and Subsequent IL-2 Dose Interruption.

A. Raw Dose-Level Counts by Post-Dose hs-Tn Threshold					
Outcome	Post-Dose hs-Tn <15 ng/L	Post-Dose hs-Tn ≥15 ng/L			Total
Clinically significant cardiac event	0/39 (0.0%)	15/48 (31.3%)			15/87 (17.2%)
No cardiac event	39/39 (100.0%)	33/48 (68.8%)			72/87 (82.8%)
Subsequent IL-2 dose interruption	5/40 (12.5%)	17/46 (37.0%)			22/86 (25.6%)
Continued planned IL-2 dosing	35/40 (87.5%)	29/46 (63.0%)			64/86 (74.4%)
**B. Clustered Logistic Regression Results**					
**Outcome**	**Predictor**	**B (SE)**	**Wald** **χ^2^**	***p***	**OR (95% CI)**
Cardiac event	Post-dose hs-Tn ≥15 ng/L	2.3 (0.940)	5.78	0.016	9.6 (1.5–60.6)
	Log post-dose hs-Tn, per 1-unit increase	—	—	0.001	3.25 (1.59–6.65)
Subsequent IL-2 dose interruption	Post-dose hs-Tn ≥15 ng/L	1.2 (0.583)	4.41	0.036	3.4 (1.1–10.7)
	Log post-dose hs-Tn, per 1-unit increase	—	—	<0.001	2.82 (1.63–4.88)

Clinically significant cardiac events were defined as QTc prolongation >480 ms, clinically significant arrhythmia excluding isolated sinus tachycardia, new electrocardiographic abnormality, vasopressor requirement, or cardiology consultation. Subsequent IL-2 dose interruption was defined as holding the next planned IL-2 dose. Odds ratios were estimated using clustered logistic regression to account for repeated IL-2 doses within patients; therefore, the model-based ORs will not necessarily match the crude 2 × 2 counts exactly. hs-Tn, high-sensitivity troponin; SE, standard error; OR, odds ratio; CI, confidence interval.

**Table 6:** Treatment-Emergent Toxicities Across the TIL Treatment Course.

Period	Toxicity	Any Grade n (%)	Grade ≥ 3 n (%)
Tumor Harvest	Allergic reaction	1 (2.8%)	0
During lymphodepleting chemotherapy	Nausea	19 (52.8%)	0 (0.0%)
	Diarrhea	14 (38.9%)	1 (2.8%)
	Fluid overload/edema	8 (22.2%)	1 (2.8%)
	Fatigue/malaise	8 (22.2%)	0 (0.0%)
	Vomiting	6 (16.7%)	0 (0.0%)
	Fever	4 (11.1%)	1 (2.8%)
	Tachycardia	4 (11.1%)	0 (0.0%)
	Dizziness	3 (8.3%)	0 (0.0%)
	Headache	3 (8.3%)	0 (0.0%)
	Hypoxia	3 (8.3%)	1 (2.8%)
	Rash/pruritus	3 (8.3%)	0 (0.0%)
	Flushing	2 (5.6%)	0 (0.0%)
	Abdominal pain	1 (2.8%)	0 (0.0%)
	Cholecystitis	1 (2.8%)	1 (2.8%)
	Encephalopathy/neurotoxicity	1 (2.8%)	1 (2.8%)
	Hyponatremia	1 (2.8%)	1 (2.8%)
	Hypotension	1 (2.8%)	1 (2.8%)
	Paresthesia/tingling	1 (2.8%)	0 (0.0%)
	Transaminitis	1 (2.8%)	0 (0.0%)
	Wheezing/shortness of breath	1 (2.8%)	0 (0.0%)
**After TIL infusion and before first IL-2 dose**	Chills/rigors	4 (11.1%)	0 (0.0%)
	Tachycardia	4 (11.1%)	0 (0.0%)
	Neutropenic fever/bacteremia	3 (8.3%)	3 (8.3%)
	Fever	2 (5.6%)	0 (0.0%)
	Flushing	2 (5.6%)	0 (0.0%)
	Hypotension	2 (5.6%)	0 (0.0%)
	Nausea	2 (5.6%)	0 (0.0%)
	Hypertension	1 (2.8%)	0 (0.0%)
	Hypoxia/desaturation	1 (2.8%)	0 (0.0%)
	Syncope	1 (2.8%)	0 (0.0%)
	Vomiting	1 (2.8%)	0 (0.0%)
	Wheezing/shortness of breath	1 (2.8%)	0 (0.0%)
**IL-2 administration**	Thrombocytopenia	32 (88.9%)	28 (77.8%)
	Neutropenic fever	21 (58.3%)	21 (58.3%)
	Transaminitis	17 (47.2%)	4 (11.1%)
	Hypophosphatemia	16 (44.4%)	4 (11.1%)
	Hypotension	5 (13.9%)	4 (11.1%)
	Acute kidney injury	4 (11.1%)	3 (8.3%)
	Hypoxia	8 (22.2%)	8 (22.2%)
	Rash	9 (25.0%)	0 (0.0%)
	Cholestasis	3 (8.3%)	0 (0.0%)
	Platelet transfusion	22 (61.1%)	—

Toxicities were graded according to the Common Terminology Criteria for Adverse Events (CTCAE), version 5.0. Percentages were calculated using the full treated cohort as the denominator (N = 36). Neutropenic fever after TIL infusion and before the first IL-2 dose included two cases with culture-positive bacteremia.

## Data Availability

The datasets generated and/or analyzed during the current study are not publicly available because of patient privacy and institutional restrictions but are available from the corresponding author on reasonable request and with appropriate institutional approvals.

## Supplementary Materials

The following supporting information can be downloaded at: https://www.mdpi.com/article/10.3390/curroncol33070379/s1, Table S1: Objective response rate by IL-2 dose category; Table S2: Kaplan-Meier estimates of overall survival and progression-free survival by subgroup; Table S3: Sample sizes for longitudinal immune reconstitution analyses; Table S4: Cardiac events during IL-2 administration (patient-level); Table S5: Association between post-dose hs-Tn and cardiac events.

## Abbreviations

The following abbreviations are used in this manuscript:

AJCC	American Joint Committee on Cancer
BOR	best overall response
BRAFi/MEKi	BRAF and MEK inhibitors
CI	confidence interval
CNS	central nervous system
CR	complete response
CRP	C-reactive protein
CTCAE	Common Terminology Criteria for Adverse Events
CTLA-4	cytotoxic T-lymphocyte-associated protein 4
DCR	disease control rate
ECOG	Eastern Cooperative Oncology Group
GEE	generalized estimating equations
HR	hazard ratio
hs-Tn	high-sensitivity troponin
IgG	immunoglobulin G
IL-2	interleukin-2
IND	Investigational New Drug
IQR	interquartile range
LDH	lactate dehydrogenase
LVEF	left ventricular ejection fraction
M1a	distant skin, subcutaneous, or nodal metastases
M1b	lung metastases
M1c	non-CNS visceral metastases
M1d	central nervous system metastases
OOS	out-of-specification
OR	odds ratio
ORR	objective response rate
OS	overall survival
PD	progressive disease
PD-1	programmed cell death protein 1
PD-L1	programmed death-ligand 1
PFS	progression-free survival
PR	partial response
RECIST	Response Evaluation Criteria in Solid Tumors
SD	stable disease
SE	standard error
TIL	tumor-infiltrating lymphocyte
ULN	upper limit of normal
