# Supplementary material for: Real-World Tumor-Infiltrating Lymphocyte Therapy for Metastatic Melanoma: Treatment Delivery, Immune Reconstitution, and Cardiac Monitoring During High-Dose IL-2
Source: Curr Oncol. 2026 Jun 24;33(7):379. doi: 10.3390/curroncol33070379 (PMC13408922; doi:10.3390/curroncol33070379)
Supplement: Supplementary file 1 [file curroncol-33-00379-s001.zip › curroncol-4323311-supplementary.pdf]

Supplementary Tables

Supplementary Table S1. Objective response rate by IL-2 dose category.

IL-2 Dose Category	N	Responders, n	ORR (%)	Non-responders, n	OR (95% CI)	*P*
≤2 doses	7	2	28.6%	5	Reference	
≥3 doses	29	16	55.2%	13	3.08 (0.51–18.54)	0.402
**Total**	36	18	50%	18		

CI, confidence interval; IL-2, interleukin-2; OR, odds ratio; ORR, objective response rate.

Supplementary Table S2. Kaplan-Meier estimates of overall survival and progression-free survival by subgroup.

Variable	Group	Median OS (months) (95% CI)	*P*	Median PFS (months) (95% CI)	*P*
**Sex**	Female	9.00 (6.34–NR)		3.25 (2.76–NR)	
	Male	NR (6.83–NR)	0.761	3.61 (2.10–5.19)	0.395
**Age at TIL**	<65 years	9.00 (6.34–NR)		3.48 (2.10–NR)	
	≥65 years	NR (7.33–NR)	0.417	3.88 (2.50–NR)	0.714
**ECOG Performance Status**	0	12.94 (7.33–NR)		3.68 (2.76–5.19)	
	≥1	3.65 (3.22–NR)	0.191	2.50 (1.38–NR)	0.606
**Melanoma subtype**	Cutaneous	13.40 (6.34–NR)		3.37 (2.50–NR)	
	Mucosal	9.00 (9.00–9.00)	0.946	3.75 (2.76–NR)	0.785
**M1d**	Yes	4.57 (3.32–NR)		2.68 (1.31–NR)	
	No	13.40 (9.00–NR)	<0.001	3.68 (2.76–NR)	0.365
**Liver mets**	Yes	13.40 (3.65–NR)		3.22 (2.50–NR)	
	No	12.94 (7.33–NR)	0.316	3.61 (2.76–NR)	0.542
**Bone mets**	Yes	7.33 (7.33–NR)		3.25 (1.25–NR)	
	No	12.94 (6.83–NR)	0.730	3.68 (2.76–NR)	0.365
**LDH > ULN**	Yes	6.83 (3.65–NR)		2.76 (1.38–NR)	
	No	12.94 (7.33–NR)	0.548	3.68 (3.22–NR)	0.160
**CRP > ULN**	Yes	9.00 (4.34–NR)		3.25 (2.76–4.07)	
	No	NR (12.94–NR)	0.079	3.94 (1.25–NR)	0.212

**Ferritin > ULN**	Yes	NR (6.83–NR)		3.88 (2.50–NR)	
	No	12.94 (7.33–NR)	0.204	3.61 (2.76–NR)	0.524
**TIL infusion to IL-2 first dose interval**	≤8 hours	9.00 (7.33–NR)		3.68 (2.76–NR)	
	>8 hours	12.94 (5.00–NR)	0.390	3.84 (2.50–2.50)	0.348
**Bridging therapy**	No	12.94 (6.83–NR)		3.94 (3.48–NR)	
	Yes	9.00 (6.34–NR)	0.787	2.76 (1.94–4.40)	0.219
**IL-2 doses**	≤2	3.65 (3.32–NR)		2.50 (0.99–NR)	
	≥3	13.40 (9.00–NR)	0.003	3.68 (2.76–5.19)	0.316

CI, confidence interval; CRP, C-reactive protein; ECOG, Eastern Cooperative Oncology Group; IL-2, interleukin-2; LDH, lactate dehydrogenase; NR, not reached; OS, overall survival; PFS, progression-free survival; TIL, tumor-infiltrating lymphocyte; ULN, upper limit of normal; OOS, out-of-specification product according to industry manufacturing standards.

Supplementary Table S3. Sample sizes for longitudinal immune reconstitution analyses.

Outcome	Baseline (M0)	Month 1	Month 3	Month 6	Total	Unique Subjects
**CD4**	20	20	16	10	66	24
**CD4 <200 (binary)**	20	20	16	10	66	24
**CD8**	20	20	16	10	66	24
**CD4:CD8 Ratio**	20	20	16	10	66	24
**IgG**	11	11	10	5	37	17

Supplementary Table S4. Cardiac events during IL-2 administration (patient-level).

Event	n (%)
**Any clinically significant cardiac event**	8 (33%)
**QTc prolongation >480 ms**	5 (21%)
**Clinically significant arrhythmia†**	3 (13%)
**New ECG abnormality**	6 (25%)
**Chest pain**	2 (8%)
**Shortness of breath**	2 (8%)
**Vasopressor requirement**	6 (25%)

**Cardiology consultation**	3 (13%)
**LVEF reduction after IL-2**	0 (0%)

†Clinically significant arrhythmia was reported as defined in the source dataset. IL-2, interleukin-2; LVEF, left ventricular ejection fraction; QTc, corrected QT interval.
Individual event categories were not mutually exclusive.

Supplementary Table S5. Association between post-dose hs-Tn and cardiac events.

A. Continuous post-dose hs-Tn levels by cardiac event status

Cardiac event	N doses	Mean rank of post-dose hs-Tn	Test	Z	*P*
No event	72	39.27	Mann-Whitney U	-3.421	<0.001
Any cardiac event	15	63.50			

B. Troponin threshold (≥15 ng/L) and cardiac events

Post-dose hs-Tn	No cardiac event	Cardiac event	Total
≤14 ng/L	39	0	39
≥15 ng/L	33	15 (31%)	48
**Total**	72	15	87

C. Diagnostic performance of post-dose hs-Tn

Outcome predicted	Sensitivity	Specificity	PPV	NPV
Clinically significant cardiac event	100.0%	54.2%	31.3%	100.0%
Next IL-2 dose interruption	77.3%	54.7%	37.0%	87.5%

hs-Tn, high-sensitivity troponin; IL-2, interleukin-2; NPV, negative predictive value; PPV, positive predictive value
